# Surface Engineered Metal-Organic Frameworks (MOFs) Based Novel Hybrid Systems for Effective Wound Healing: A Review of Recent Developments

**DOI:** 10.3389/fbioe.2020.576348

**Published:** 2020-09-17

**Authors:** Luo-Qin Fu, Xiao-Yi Chen, Mao-Hua Cai, Xiao-Hua Tao, Yi-Bin Fan, Xiao-Zhou Mou

**Affiliations:** ^1^Department of General Surgery, Chun’an First People’s Hospital (Zhejiang Provincial People’s Hospital Chun’an Branch), Hangzhou, China; ^2^Key Laboratory of Tumor Molecular Diagnosis and Individualized Medicine of Zhejiang Province, Zhejiang Provincial People’s Hospital, People’s Hospital of Hangzhou Medical College, Hangzhou, China; ^3^Clinical Research Institute, Zhejiang Provincial People’s Hospital, People’s Hospital of Hangzhou Medical College, Hangzhou, China; ^4^Department of Dermatology, Zhejiang Provincial People’s Hospital, People’s Hospital of Hangzhou Medical College, Hangzhou, China

**Keywords:** metal-organic frameworks, surface modification, wounds healing, bactericidal, dressings

## Abstract

Wounds present serious medical complications and their healing requires strategies that promote angiogenesis, deposition of collagen as well as re-epithelialization of wounds. Currently used conventional wound healing strategies have become less effective due to various issues associated with them. Thus, novel strategies are needed to be developed for early and effective healing of wounds. Metal-organic frameworks (MOFs), formed by linking of metal ions through organic bridging ligands, are highly tunable hybrid materials and have attracted more considerable scientific attention due to their charming and prominent properties, such as abundant pore structures and multiple functionalities. Surface engineering of MOFs with unique ligands can overcome issues associated with conventional wound healing methods, thus resulting in early and effective wound healing. This review has been undertaken to elaborate wound healing, and the use of surface engineered MOFs for effective and rapid wound healing. The process of wound healing will be discussed followed by a detailed review of recent literature for summarizing applications of surface engineered MOFs for wound healing. MOFs wound healing will be discussed in terms of their use as antibacterial agents, therapeutic delivery vehicles, and dressing systems in wound healing.

## Introduction

Skin is the largest organ of the human body that protects the body against external threats, such as injury and microbial attack (Xu et al., 2015). However, skin damage is one of the most common everyday emergencies and could severely affect the health and lives of people, especially when the defect is severe (Admassie et al., 2018). Wounds are usually considered painful injuries of the skin tissue, when dermal layers are cut, punctured, or broken due to external stimulus or damage. In everyday life, wounds are a complex and challenging clinical problem linked to numerous complications that lead to frequent mortality and morbidity (Natarajan et al., 2000). Wounds are typically categorized into two categories as per their time frame of healing. Wounds that get cured in a predictable period followed by anatomical and functional restoration of tissue are called acute wounds (Harding et al., 2002). Such wounds are usually caused by severe tissue damage or surgery. On the other side, wounds that do not cure in a consistent period contributing to further problems, such as inflammation and healing issues are classified as chronic wounds. Chronic wounds include vascular ulcers, pressure ulcers, and diabetic ulcers (Nethi et al., 2019).

Cutaneous injuries are a global part of medical care, with around 300 million severe patients around the world and 100 million traumatic wound patients. Wounds have an enormous financial burden on healthcare systems globally, representing more than $25 billion annually in the US alone (Sen et al., 2009; Das and Baker, 2016). The wound care products market has expanded massively to over US$ 15 billion, including US$ 12 billion to deal with wound scars (Nethi et al., 2019). Unexpectedly, healthcare providers and patients in the US are charged a cost of around US$ 9 billion annually to perform lower limb amputations resulting from diabetic foot ulcers (Falanga, 2005). The aging population is at heightened risk of the socio-economic and medical pressures induced by complications of wounds (Nethi et al., 2019). Such high health and socio-economic pressures reinforce the need for intensive work to create new therapies in this area.

The process of repairing and regenerating wound tissue is among the most complicated biological mechanism in humans, involving angiogenesis, tissue remodeling, cell proliferation, and others (George Broughton et al., 2006; Qiu et al., 2020). Numerous treatments have also been tested with positive findings to preserve safe skin and prolong the wound healing cycle. Wounds may be handled with conventional as well as new therapies. Fascinating alternatives are conventional therapies that employ larvae, plant extracts, maggots, honey, and propolis. Such conventional medicines are commonly used globally, in particular in Latin America, Asia, and Africa (Pereira and Bartolo, 2016). Besides conventional therapies, there are numerous new approaches. Hyperbaric oxygen therapy is one of the latest wound healing approaches that use oxygen flows to the site of injury and finally retards amputation (Santema et al., 2018). Negative pressure wound therapy is used for various types of both chronic and acute wounds. It helps to improve angiogenesis by discarding interstitial fluids and bacterial infections at the site of the wound and clearing lymph nodes (Armstrong et al., 2002; Schurtz et al., 2018). Currently, bioengineered cell construct has become of paramount importance in the management of venous leg ulcers, which are a form of a life-threatening chronic wound with a high degree of morbidity (Stone et al., 2017). Dressing of polymer-containing materials that imitate the physical and biological properties of the surrounding tissues ultimately helps the process of healing (Pereira and Bartolo, 2016). Surgery is still very a popular treatment for untreated wounds that are not healed (Thakral et al., 2015).

Conventional wound treatment methods have been associated with some drawbacks, such as the occurrence of allergic reactions, uncertain mechanisms of action, possible contamination, fast drying of the wound region, and variability in batch to batch (Walgrave et al., 2005; Jurczak et al., 2007). Medications utilized for wound treatment have certain limitations including contribution to paranoia, peptic ulceration, hyperglycemia, and slow healing of bones. Other limitations include inhibition of the healing process by decreasing cell mobility to injury sites, the effect on collagen operation, hampering fibroblasts formation, minimizing oxygen flow to the wound site, and reducing immunity which delays the healing of wounds (Ovais et al., 2018). Various other factors, such as malnutrition, obesity, diabetes, medication, and lifestyle deregulate the process of wound healing by extending or stopping incidents in any phase of wound healing (Anderson and Hamm, 2012). Also, bacterial infection and the slow separation of fibroblasts may hinder the healing process, contributing to swelling, local pains, and even thus threatening complications (Lai and Rogach, 2017; Ren et al., 2019). Thus, there is a critical need to build alternative solutions to address unmet health challenges involved in wound care management (Nethi et al., 2019). Nanotechnology is an increasingly growing scientific area and has been used to solve various biological issues, both therapeutic and diagnostic. Numerous nanotechnologies have appeared over the last few decades, creating enormous possibilities because of their unique properties and perfect applications. These technologies can address the complexity of the wounds and their specificity. Such nanotechnologies include the use of numerous metal-dependent nanoparticles and metal-organic frameworks (MOFs) (Hamdan et al., 2017; Ren et al., 2019).

Metal-organic frameworks (MOFs) are among nanotechnology’s most exciting and high-profile areas that have appeared in the last decade (Hinks et al., 2010). MOFs are a group of crystalline coordinating materials made up of metal ions or clusters widened of organic polydentate ligands (Yu et al., 2018). The term MOFs was coined in 1995, and the filed gained momentum in 1999 after the work of Chui et al. (1999). MOFs have been thoroughly researched for applications in various areas including chemistry, climate, energy, and biomedicine, due to their unique properties such ultra-high surface area, tuned but consistent pore sizes, strong thermal stability, large internal spaces, biocompatibility, and easy synthesis (Yu et al., 2017, 2018). In recent years, MOFs have gained more interest in the field of biomedical applications, such as drug carriers, biological imaging, sensing, and theranostic nanovectors due to their unique benefits of large pores, biodegradation, biocompatibility, increased drug loading, and flexible size (Yu et al., 2017). Currently, MOFs are getting wider attention for wound healing owing to their increased drug loading, surface modulation for targeted delivery and controlled released of the wound healing agents, lesser toxicities, intrinsic angiogenic and antibacterial properties as compared to other nanomaterials (Xiao et al., 2017; Chen et al., 2019). As compare to other nanomaterials, MOFs have metals ions, for instance, copper, as their central structural blocks. Thus, these central blocks of MOFs are intrinsically involved in various wound healing processes, such as angiogenesis, promotion of vascular endothelial growth, and stabilization and expression of various extracellular skin proteins including collagen and keratin (Xiao et al., 2017, 2018; Kargozar et al., 2019b). Furthermore, certain elements used as structural blocks of MOFs, for instance, zinc and copper, help in wound healing of infectious wounds of *S. aureus* and *E. coli* due to their natural bactericidal properties, thus leading to effective and rapid wound healing. Similarly, MOF-based wound dressing accelerates wound healing due to its bactericidal and anti-inflammatory properties through encouraging cell proliferation, angiogenesis, and collagen deposition (Hinks et al., 2010; Rubin et al., 2018; Hou and Tang, 2019; Kargozar et al., 2019a; Yao et al., 2020). Interestingly, MOFs have free functional groups decorated on their surfaces, thus they provide opportunities for their surface functionalization. Surface engineered MOFs render unique properties, such as targeted delivery, stability, controlled release of their loaded contents, and additional biological properties (Ghaffar et al., 2019). Recently surface engineered MOFs have attracted a greater scientific interest for wound healing due to their intrinsic biological properties and delivery and controlled release of therapeutic agents (Zhang M. et al., 2020; Zhang S. et al., 2020). Moreover, the concept of surface engineered MOFs systems with or without therapeutic agents is gaining increasing popularity for quick and cost-effective wound healing (Yu et al., 2018; Ren et al., 2019; Zhang M. et al., 2020).

To the best of our knowledge, no review has been published on applications of surface engineered MOFs for effective wound healing. This review has been undertaken to highlight the applications of surface engineered MOFs for effective and early wound healing. The process of wound healing will be discussed followed by a detailed literature review for summarizing applications of surface engineered MOFs for wound healing. MOFs wound healing will be discussed in terms of their use as antibacterial agents, therapeutic delivery vehicles, and dressing systems in wound healing. This review will be an excellent addition to the body of scientific knowledge for understanding applications of MOFs for early and effective wound healing.

## Process of Wound Healing

**FIGURE 1:**
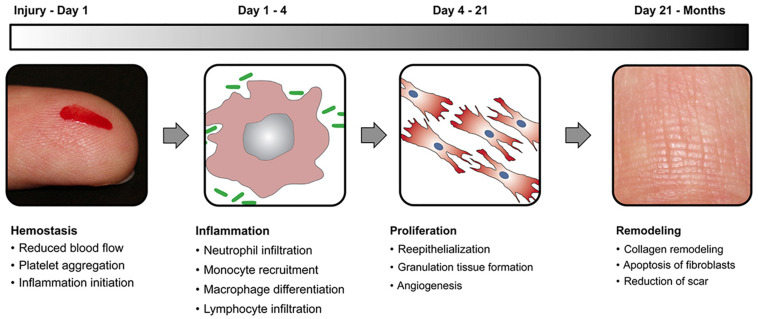
A schematic representation of wound healing stages with a time scale for each stage. Reproduced from Das and Baker (2016).

Wound healing is a natural physiological process that takes place in reaction to any damage or injury to the tissue. The process includes complex interactions between different types of cells, factors of coagulation, connective tissue, growth factors, cytokines, and the vascular system. Four steps in the wound healing cycle are illustrated in Figure 1. The hemostasis is the very first stage in wound healing. After the initial wounding, the bleeding occurs and hemostasis is needed. During hemostasis, bleeding is terminated by the process of platelet aggregation, vasoconstriction, and blood coagulation. The next stage in the healing process is inflammation that is triggered by the inflammatory mediators (prostaglandins and histamine), which increases the nearby vessel’s permeation and vasodilation. Inflammation lasts for 4 days, and during this process, immune cells are recruited to reduce infection and stimulate capillary growth. After Inflammation, the next stage is the proliferative phase, in which the wound area is created with fresh collagen- and extracellular matrix (ECM) connective tissue. Myofibroblasts assemble across the wound margin and draw the edges via contracting, which facilitates the healing process rapidly (Brown et al., 1988; Naseri et al., 2017). The last stage of the proliferation process is re-epithelialization, wherein epithelial cells expand and migrate through the wound. A moist wound atmosphere speeds this cycle up. This stage of wound healing may last for several months and is characterized by the continuous low rate of collagen degradation, synthesis, and reorganization. In compromised patients, various factors lead to poor or slow healing, thus this in turn results in the development of chronic wounds. Examples of such wounds are ulcers in diabetic patients (Junker et al., 2013).

### Surface Engineered MOFs in Wound Healing

In the current decade, substantial progress has been made in the widespread usage of MOFs in the field of pharmaceutical therapy, owing to their well-defined porous aperture, fast drug loading, increased medication bioavailability, and long operation duration (Li et al., 2017; Wu and Yang, 2017; Abánades Lázaro et al., 2018). The high structural designability and flexible functionality of the MOFs in specific create synergistic effects with the drugs, helping to make them potential candidates for healing multiple kinds of wounds (Liu et al., 2019; Zhang et al., 2019b). MOFs are widely used for wound healing owing to their increased drug loading, surface modulation for targeted delivery and controlled released of the wound healing agents, lesser toxicities, intrinsic angiogenic and antibacterial properties as compared to other nanomaterials (Xiao et al., 2017; Chen et al., 2019). The wound healing properties of MOFs can be further improved through their surface engineering. Interestingly, MOFs have free functional groups decorated on their surfaces, thus they provide opportunities for their surface functionalization or engineering. Surface engineered MOFs render unique properties, such as targeted delivery, stability, controlled release of their loaded contents, and additional biological properties (Ghaffar et al., 2019; Zhang S. et al., 2020). Many functional biological molecules can be chemically or physically conjugated or adsorbed on the surfaces of MOFs via surface engineering, thus leading to synergistic properties. Furthermore, surface engineered MOFs render improved sustain release properties, therefore, they efficiently maintain the therapeutic concentration of their loaded drugs for a longer time (Meng et al., 2020). Applications of surface engineered for wound healing can be discussed under the following sub-headings.

### Bactericidal Surface Engineered MOFs in Wounds Healing

Among the complexities, the bacterial infection has become a major issue that has a major impact on normal skin healing (Zhang et al., 2019a). Metallic antibacterial agents, such as zinc, silver, copper-based oxides, or ions have been employed for the last few decades to kill or inhibit the growth of bacteria across the wounds (Chen et al., 2018). Nevertheless, the usage of metallic antibacterial agents is also dangerous because of their possible toxicity caused by the sudden release of metal ions (Dreifke et al., 2015). Hence there is a considerable therapeutic need to alleviate the toxic effects of metallic antibacterial agents by delaying the discharge of metal ions. Some scientists have previously shown the antibacterial ability of MOFs that lead to their slow release of metal ions (Zhuang et al., 2011; Lu et al., 2014). Because of its adjustable chemical and physical properties, the release of metal ions from MOFs becomes suitable for fine-tuning, making MOFs suitable for wound dressing (Zhang et al., 2014; Mason et al., 2015).

**FIGURE 2:**
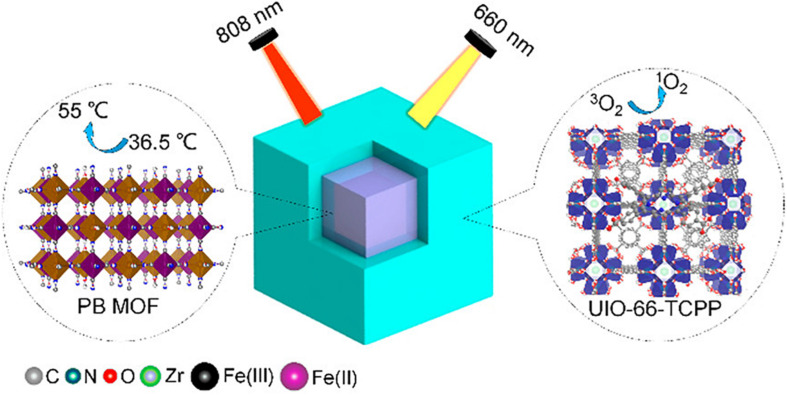
Schematic presentation of the core-shell structure of UIO-66 MOFs. The surface engineered MOFs contain Prussian blue as a core and porphyrin as a shell for simultaneous PDT and PTT therapies against bacteria in wound healing. Used with permission from Hou and Tang (2019). Note to readers: Further permissions related to the material excerpted should be directed to the ACS).

In a recent study, Luo and colleagues developed versatile surface engineered MOFs systems with properties of both photodynamic therapy (PDT) and photothermal therapy (PTT). The novel system was highly efficient in achieving antibacterial activity against bacterial species in wound infections. UIO-66 MOFs were synthesized and were surface engineered in a way that they contained Prussian blue (used for PTT) in core and porphyrins (used for PDT) as a shell. This core-shell PB@MOF not just to acquires Prussian blue’s photothermal influence (Figure 2) but also attaches porphyrin’s photodynamic value, thereby demonstrating an outstanding synergistic activity toward bacterial infection. Those versatile MOFs demonstrated good activity toward *S. Aurores* and *E. Coli*, the bacterial genus most widespread in infections, under optimization situations of PTT and PDT. They further researched the synthesized MOFs for their extracorporeal wound healing infection of rat form *S. aureus*. After 14 days of therapy, full wound healing was observed without injury in the key rat organs, demonstrating the health of dual surface-engineered MOFs (Hou and Tang, 2019).

A recent study reported the fabrication of a powerful surface-adaptive, on-demand antimicrobial nanoplatform using surface engineered MOFs for effective healing of the infectious wound. Photo-sensitive MOFs were surface-functionalized with hyaluronic acid (HA) followed by their loading on Ag ions. The novel multi-functional MOFs showed good biocompatibility with non-targeted cells, as negatively charged HA prevented the release of Ag ions. While in the presence of targeted bacteria, the secreted hyaluronidase degraded HA on and produced positively charged nanoparticles. This in turn increased the MOFs affinity toward bacteria and resulted in a synergistic antibacterial effect due to the released Ag ions and generation of reactive oxygen species under visible light irradiation. When further studied *in vivo*, the novel multi-functional MOFs showed significant impacts on the treatment of multidrug-resistant wounded bacteria in mice models (Zhang et al., 2019b). In a similar study, silver nanoparticles of ultra-small size were synthesized using γ-cyclodextrin MOFs. These MOFs embedded with silver nanoparticles exhibited increased bacterial inhibition. GRGDS peptide, which promotes hemostatic plug formation through binding to integrin GPIIb-IIIa receptor on activated platelets was used for further surface functionalization of these MOFs. These surface-functionalized MOFs showed elevated wound healing through hemostatic effects in synergy with the antibacterial effect of silver nanoparticles embedded in MOFs (Shakya et al., 2019).

In another recent study, surface engineered niacin MOFs were fabricated with alginate shell and copper-/zinc-niacin framework cores and used for wound healing. The niacin MOFs were having outstanding properties of angiogenesis, antioxidant, and antibacterial. The alginate shells of the smart MOFs were bacteria responsively degradable. The niacin MOFs were capable of releasing copper, zinc, and calcium ions in an intelligent, controllable, and programmable manner in response to the degree of infections. The released ion destroyed infectious microbes through their membrane destruction and induced the outflow of nutrient substances. They also activated copper/zinc superoxide dismutase (Cu/Zn-SOD) to eliminate oxygen free radicals and rescuing the cells from an oxidative stress injury. Furthermore, the simultaneously released niacin promoted hemangiectasis and the absorption of functional metal ions. Additionally, the continuously generated niacin facilitated hemangiectasis and uptake of functional metal ions. These MOFs further improved the healing process of the wound of the injured full-thickness skin defect model (Chen et al., 2019).

### Surface Engineered MOFs in Multi-Functional Dressings for Effective Wound Healing

During the healing process, the tissue must be protected from infectious diseases and other environmental external damage (Sen et al., 2009). Wound dressings are preferred for protecting wounds from bacterial colonization and other environmental hazards. Distinct wound dressings were required to fight bacteria by embedding bactericidal ingredients like polymeric substances and metal ions (Yao et al., 2020). The dressings are also made from inherent hydrophilic fabrics, like cotton, polymers, and hydrogels, to promote the infiltration of released active ingredients into wounds (Dhivya et al., 2015). Nevertheless, many other hydrophilic dressings have tiny evident contact angle (CA); elevated biofluids could be strongly stuck to the rough matrix of the dressing after they have been wet (Shi et al., 2019). Unabsorbed biofluids can overhydrate the wound that can cause vulnerability to microbial invasion, tissue edge maceration, and extracellular matrix damage, bacterial colonization, and tissue damage (Okan et al., 2007; Wild et al., 2010). Therefore, the development of new and multifunctional protective wound dressings is urgently needed. Highly porous MOFs surface modified with functional bactericidal materials is currently getting increasing interest for the fabrication of effective and safe wound dressing materials. Being intrinsically bactericidal, further surface modification of MOFs can synergistically improve their wound healing activity. Furthermore, they can also be loaded with therapeutic substances and release them in a programmable manner, thus leading to effective wound healing with higher safety (Xiao et al., 2017; Yao et al., 2020).

Most recently, Yao et al. reported the fabrication of omniphobic MOFs based hydrogel porous wound dressing for inhibition of bacteria invasion and acceleration of wound healing. The novel MOFs based hydrogel showed a controlled release of the non-toxic zinc ions anti-inflammatory and bactericidal. Besides, the wound dressing relying on MOFs has speeded up the wound healing in an infected full-thickness skin defect model due to angiogenesis, synergistic antibacterial effects, reduced inflammation, the proliferation of fibroblasts and collagen deposition (Yao et al., 2020). In another most recent study, Han et al. (2020) synthesized photosensitive wound dressing hydrogels using MOFs modified with double-bond modified chitosan, Prussian blue, and quaternary ammonium. The synthetic hydrogels showed excellent photothermal characteristics under near-infrared light irradiation of 808 nm. MOFs based novel hydrogel was capable of capturing bacteria tightly through electrostatic interactions due to the presence of chitosan. The presence of bactericidal chitosan and photothermal Prussian blue in the MOFs based hydrogel synergistically increased its antibacterial effects. When used as a wound dressing for wound cuts in rates, the MOFs based hydrogel demonstrated increased wound healing without causing injuries in other organs (Han et al., 2020).

**FIGURE 3:**
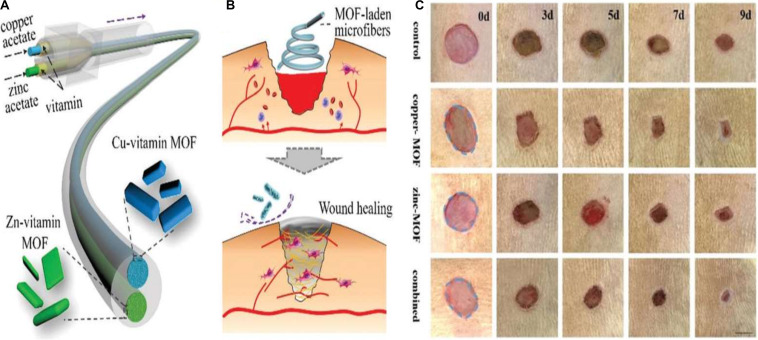
**(A)** Schematic presentation for vitamin MOF-laden hydrogel microfibers fabrication, **(B)** Schematic presentation for wound healing applications of the fabricated vitamin MOF-laden the application of the microfibers in the wound healing process, and **(C)** Representative photos of the skin wounds treated with fabricated MOFs systems. Reproduced from reference Yu et al. (2018) permission conveyed through Copyright Clearance Center, Inc.

**FIGURE 4:**
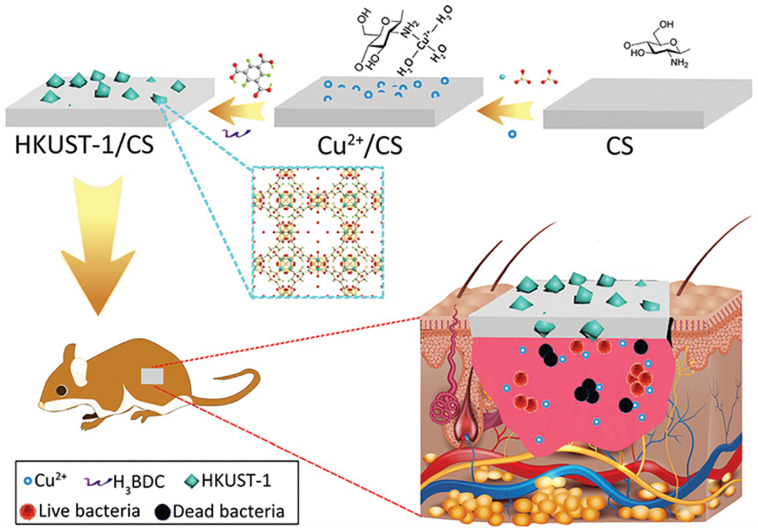
Schematic presentation of the novel MOFs based multi-functional films and their *in vivo* wound healing application. Used with permission of Royal Society of Chemistry, from “Copper metal–organic frameworks loaded on chitosan film for the efficient inhibition of bacteria and local infection therapy. Permission conveyed through Copyright Clearance Center Ren et al. (2019).

The application of copper ions has already shown potential likely by encouraging angiogenesis in wound healing applications. Copper is a well-known antimicrobial agent and can significantly enhance healing by lowering the risk of wound infection. Even so, identifying treatment options using copper ions involves additional copper salts or oxides to be applied to the wound site, subjecting the patient to highly harmful levels of copper ions and leading in inconsistent effects (Mandinov et al., 2003; Guo et al., 2013; Amna et al., 2014). The toxicity can be mitigated, though, when copper ions are gradually emitted from a repository at the wound. Xiao et al. developed a highly safe cooperative cooper MOFs that were surface engineered through their embedding into an antioxidant thermoresponsive citrate-based hydrogel for accelerated wound healing in diabetic mice. These smart surface engineered cooper MOFs hydrogel showed significantly accelerated wound healing with higher safety owing to their controlled release capability of copper ion (Xiao et al., 2017). A recent study by Yu et al. (2018) reported the fabrication of copper- or zinc-MOF-laden hydrogels containing vitamin for effective wound healing. The novel MOF-laden hydrogel microfibers of alginate shells and copper- or zinc-vitamin frame cores use a microfluidic coaxial capillary spinning method (Figure 3). Due to the antioxidation and antibiosis features of their controllably released vitamin ligands, zinc ions, and copper ions, the novel MOFs system demonstrated the practical value in tissue wound healing (Yu et al., 2018). A similar approach for fabrication of effective wound dressing was adopted by Ren et al. They synthesized copper-based MOFs loaded with chitosan-based multi-functional films (HKUST-1/CS) for effective antibacterial and wound healing purposes. Due to the slow release of copper ion and the presence of high contents of chitosan in these MOFs, an improved antibacterial activity against *S. aureus* and *E. coli* was achieved. Furthermore, novel MOFs based these multi-functional films effectively prevented *S. aureus* bacteria-derived wound infection (Figure 4) and enhanced the cutaneous wound repair without recurrence by stimulating the formation of blood vessels, epidermis and dermis (Ren et al., 2019).

In another similar recent study, Wang et al. copper-based MOFs surface modified with chitosan/polyvinyl alcohol fibers as a wound dressing for enhanced antibacterial and wound properties. The MOFs based novel dressing materials showed increased antibacterial against *S. aureus* and *E. coli*, the most common bacterial species in wound infections. In animal studies, contrasted with commercial dressings of chitosan and chitosan/PVA fibers, these MOFs surface modified with fibers were more potent to heal the wound with minimum inflammation (Wang et al., 2020). In another recent study, Yang et al. synthesized surface engineered MOF derived photo-responsive nanocarbon for safe, rapid, and synergistic chemophotothermal bacterial disinfection of wounds. The novel surface engineered MOFs demonstrated nearly 100% bactericidal activity at a very low dose. They also particularly efficient and healthy disinfection of skin wounds that were comparable with vancomycin (Yang et al., 2019).

### Surface Engineered MOFs in Therapeutics Delivery for Wound Healing

Owing to their porous nature and large surface area, MOFs are used as depot systems for loading and delivering therapeutic agents for wound healing. Studies have recently identified that NO plays a key role in the wound repair process, demonstrating that growing endogenous NO synthesis or exogenous NO can inhibit inflammation, promote angiogenesis and enhance collagen deposition throughout the healing process (Zhou et al., 2017; Champeau et al., 2018). To obtain an effective NO-based therapy for diabetic wound healing, however, regulating the NO-release activity and preserving its optimal concentration is essential. Owing to their unique properties including, porosity and extreme-high specific surface area, adjustable particle size, easy to be functionalized, and low skeleton density, MOFs have emerged versatile carriers for NO delivery for effective wound healing. Also, due to their more active sites that can sustainably incorporate with more gas molecules, MOFs show admirable superiority in delivery and gas storage (Furukawa et al., 2013; Li et al., 2016). Within the MOFs, in particular, there are many coordinately unsaturated metal sites (CUS) that can establish close relationships with NO. This ability makes the MOF a successful vehicle for NO storage applications for permanent and long shelf life (Hinks et al., 2010).

Diabetic wounds are serious complications associated with diabetes. Their healing process is extremely slow or they even do not heal. Researchers have found that high sugar contents prevent the production of endogenous NO, thus inhibit the healing of diabetic wounds (Schäffer et al., 1997; Luo et al., 2004). Thus, externally applied NO has become an effective therapeutic strategy for quick and effective healing of diabetic wounds. In a most recent study, copper-based core-shell MOFs surface engineered with 4-(Methylamino) pyridine and prepared through the electrospinning method was reported as a NO-loading and sustained release system for enhanced diabetic wound healing (Zhang P. et al., 2020). The surface engineered MOFs system revealed long release and half-life storage stability, higher biodegradability, and biocompatibility. Furthermore, copper ions and NO synergistically stimulated angiogenesis, promoted collagen deposition and inhibited inflammation in the wound area, thus resulting in complete healing of the diabetic wound in 2 weeks in mice.

In another recent study, Li et al. designed and developed cobalt-based nano MOFs (ZIF-67) as carriers for loading and delivering a pro-angiogenic drug dimethyloxalylglycine for long-term therapy of diabetic wound. The drug-loaded MOFs were further surface engineered into a dual cooperative controllable release system through their incorporation into micro-patterned PLLA/Gelatin nanofibrous scaffolds. The novel system was capable of releasing the loaded drug as well as copper ions continuously for more than 15 days. *In vitro* study has demonstrated that the released copper ions and drug from the novel surface engineered MOFs system could therapeutically enhance the migration, proliferation, and tube formation of the human umbilical vein endothelial cells by upregulating the expression of angiogenesis-related genes and triggering a hypoxia response. Besides, the *in vivo* findings showed that the latest surface built MOFs system could substantially enhance angiogenesis, collagen deposition, and reduce inflammation in diabetes wounds (Li et al., 2020). In another study, copper-based MOFs nanoparticles were synthesized and were further modified with folic acid. Surface modification of MOFs with folic acid not only reduced the copper-related toxicity but also significantly accelerated the wound healing process in diabetic mice (Xiao et al., 2018).

## Conclusion and Future Perspectives

Wounds not only present severe medical complications but also lead to an enormous financial burden on healthcare systems globally. Currently used conventional wound healing strategies have become less effective due to various issues associated with them. Researchers are exploring alternative that is highly safe, effective, and result in early wound healing. MOFs are highly tunable hybrid materials and have recently attracted greater scientific attention for effective wound healing due to their prominent properties. Surface engineering of MOFs with unique functional materials can overcome issues associated with conventional wound healing methods, thus resulting in early and effective wound healing with higher safety. Our survey of the literature shows that, during the last 3 years, surface engineered MOFs have been reported for effective wound healing. They have been reported as antibacterial agents, therapeutic delivery vehicles, and dressing systems in wound healing. Such surface-modified MOFs accelerated wound healing through their inherent bactericidal properties, releasing their contents in a controllable manner and aiding to physiological processes of wound healing. Being a new area of research, all the studies have been carried out *in vitro* or *in vivo* in animal wound models. Yet no surface-modified MOFs system has been entered clinical studies. Having versatile structures and being capable of functionalization with multiple active molecules, they are expected to be promising candidates for wound healing shortly. However, this will require the researchers to explore them in more advanced levels.

## Author Contributions

LQF and XYC wrote and collected the data. MHC and XHT arranged the data and designed the figures. YBF and XZM revised the manuscript and designed and supervised the whole study. All authors contributed to the article and approved the submitted version.

## Conflict of Interest

The authors declare that the research was conducted in the absence of any commercial or financial relationships that could be construed as a potential conflict of interest.
